# Speech emotion recognition via graph-based representations

**DOI:** 10.1038/s41598-024-52989-2

**Published:** 2024-02-23

**Authors:** Anastasia Pentari, George Kafentzis, Manolis Tsiknakis

**Affiliations:** 1 Institute of Computer Science, Foundation for Research and Technology-Hellas, Heraklion, GR-700 13 Greece; 2 Computer Science Department, University of Crete, Heraklion, GR-700 13 Greece; 3 Department of Electrical and Computer Engineering, Hellenic Mediterranean University, Heraklion, Greece

**Keywords:** Engineering, Mathematics and computing

## Abstract

Speech emotion recognition (SER) has gained an increased interest during the last decades as part of enriched affective computing. As a consequence, a variety of engineering approaches have been developed addressing the challenge of the SER problem, exploiting different features, learning algorithms, and datasets. In this paper, we propose the application of the graph theory for classifying emotionally-colored speech signals. Graph theory provides tools for extracting statistical as well as structural information from any time series. We propose to use the mentioned information as a novel feature set. Furthermore, we suggest setting a unique feature-based identity for each emotion belonging to each speaker. The emotion classification is performed by a Random Forest classifier in a Leave-One-Speaker-Out Cross Validation (LOSO-CV) scheme. The proposed method is compared with two state-of-the-art approaches involving well known hand-crafted features as well as deep learning architectures operating on mel-spectrograms. Experimental results on three datasets, EMODB (German, acted) and AESDD (Greek, acted), and DEMoS (Italian, in-the-wild), reveal that our proposed method outperforms the comparative methods in these datasets. Specifically, we observe an average UAR increase of almost $18\%$, $8\%$ and $13\%$, respectively.

## Introduction

Speech emotion recognition (SER)^1^ is an area of research which has gained attention as a powerful tool in many fields, especially including healthcare assistance and human-robot interaction^2^. Many researchers have addressed the problem of revealing the emotions from speech signals, by exploiting a variety of speech characteristics, with the most prominent being pitch, energy, jitter and shimmer, loudness, and other spectral or time domain measures, thus creating valuable sets of speech-based features^3,4^. Among the most well-established feature sets are the Geneva Minimalistic Acoustic Parameter Set (GeMAPS or eGeMAPS)^5^, the Interspeech sets^6,7^, and the openSMILE^8^ feature set. Although these feature sets have been proved effective on many datasets, the SER problem still remains, due to speech dissimilarities among speakers, datasets, languages, and cultures^2^.

More recently, deep learning architectures have been introduced to this area, significantly complementing the conventional ML approaches^5,9^. Although these attempts are really promising, their main disadvantages are that they not only require a significant amount of data to train their models, which further increases the computational cost^2^, but also do not provide significant information about the characteristics of speech and the qualitative differentiations among the expressed emotions. As a consequence, due to computational and big data constraints on the one hand^9^, and the large variety of speech-based characteristics which create impractical high-dimensional feature spaces^10^ on the other, there is an increasing demand of addressing the SER problem from different perspectives. On the other hand, a common problem in the datasets’ analysis and classification is their imbalance, a problem that relates to both the length of speech signals as well as to the number of the available utterances per emotion. The imbalance problem can affect the performance of the experimental results and lead to biased classification accuracies^11^.

Graph-based theory has been proved a powerful tool to signal processing, as the graph representations have the ability to exploit the interrelations among the signals or segments of signals^12^. Hence, graphs can be based on the statistical information of the signals or even their structural one, through a variety of approaches including the Pearson’s correlation and the visibility graph theory, respectively. Lately, the graph-based theory was introduced to SER field, by exploiting the structural information and it was proved really promising^13^. Thus, in this study we aim to extend this previous work and provide a novel pipeline to analyze appropriately the speech signals.

In graph theory for time series, the most important quantity is the *adjacency matrix*^14^. The adjacency matrix is a square matrix denoting the interdependencies among the quantities that are compared. In our computational pipeline, we aim to exploit both the statistical and structural information, thus creating *two* different adjacency matrices. The main approach of constructing these matrices is based on taking specific mathematical relations among segments of speech signals (of fixed as well as varying length). In this work we aim to extend our previous work proposed in^13^. That approach was the first evaluation of whether graph-based theory could be proved effective towards the SER problem. Our results showed that the exploitation of the structural speech information as time series can provide an important perspective of addressing this problem.

The main contributions of this work are the following:

- We extract and exploit well-known graph-based features derived from two different adjacency matrices, constructed from two unique and novel pipelines, the one was based on the structural information whereas the other on the statistical information of the speech signals.
- Instead of retaining the whole number of utterance-based features, we take the statistical metrics of the mean value, standard deviation, kurtosis and skewness over the number of the utterances concerning each emotion per speaker. This led to a denoted as speaker-based motif classification of the available data.
- Finally, the evaluation of our proposed methodology was based on actor-based as well as in-the-wild public databases.

To the best of our knowledge, this is the first advanced graph-based pipeline to the analysis of speech signals and further, the recognition of the expressed emotions.

The rest of the paper is organized as follows: Section "Related work" discusses related work on SER. Section "Methodology" introduces our proposed methodology which includes the graph-based information presentation, the graph-based features extracted and concludes with the speaker-based approach. The experimental evaluation is presented in Section "Experimental evaluation" and in Section "Discussion" we discuss and compare our proposed methodology with the existing literature. Finally Section "Conclusions" concludes the paper and discusses future research directions.

## Related work

First attempts towards emotion recognition from speech led to representing emotional speech with a set of features, suggesting that the emotional content can be encoded in numerical values and their variation. Among the most important sets of features are the Interspeech^6,7^, the GeMAPS or eGeMAPS^5^, and the openSMILE^8^ feature set. All of them include a variety of existing speech-based features. These sets were combined with well-known classifiers such as the support vector machine (SVM)^15^, hidden Markov models (HMM)^16^, Gaussian mixture models (GMM)^17^, and others^3^. Although the feature extraction part has been proved to be robust, especially when using the GeMAPS feature set^5^, the complexity of speech emotion recognition made researchers emphasize on alternative approaches, and more specifically in the introduction of deep learning (DL) architectures. Among the most effective procedures include the analysis of speech using time and frequency information that is successively fed to Convolutional Neural Networks (CNNs)^18–20^. Inspired by the promising results, researchers introduced transfer learning in speech emotion recognition, i.e., they used a Residual Network (ResNet) pre-trained on large amount of emotional speech databases and tested on other databases^9^. Other recent works are formulated both on DL architectures, such as attention mechanisms^21^ and advanced LSTM architectures^22^, and on feature extraction, such as phase information^23^ and mel-frequency magnitude coefficients (MFMC)^24^, showing that SER is still a highly active research area. Finally, important contributions to the SER problem have been proposed in the studies^25,26^.

Recently, the Transformer model^27^ has been applied on the SER field and has gained the researchers’ interest. In the past, researchers aimed to solve the problem of the heterogeneity of data from different modalities by multimodal emotional representations through cross-transformer encoders composition^28^. Moreover, the swin-transformers were combined with the traditional spectrogram-based SER analysis in^29^. In addition, a multi-scale temporal transformer analysis achieved in^30^, providing beneficial capabilities to the SER field. Overall, without overlooking the remarkable SER results, transformer-based analyses proved to be computationally demanding and more appropriate for multilingual and multimodal SER analysis, as in^31–33^.

## Methodology

In this section we aim to analyze the main building blocks of our proposed methodology. Specifically, our speech analysis relies on the graph-based theory, including structural and statistical information of a time series. Thus, we firstly introduce the extraction of the structural graph-based speech representation through the Visibility Graph (VG) theory^13^. After that, we extend our description to the statistical information computational approach. Having denoted these two directions from the graph-based perspective, we move on to the analysis of the graph-based features, used in terms of our study. Finally, the last part of this section concerns the speaker-based model definition, i.e., we present the first four probabilistic moments used to describe the unique feature identity of each emotion per speaker.

### Structural Graph-based Speech Information

**Figure 1:**
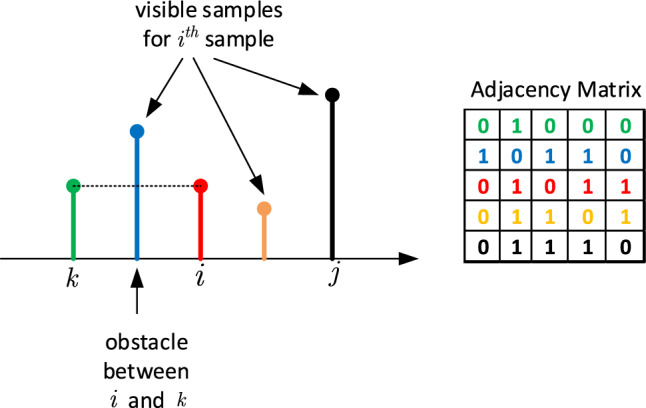
An example of the visibility graph theory and the construction of the adjacency matrix from samples of a time series.

In computational geometry, the so-called Visibility Graph (VG) theory is a simple and fast tool for converting a *positive-valued* time series into a graph^34^. The extracted graph denotes the inter-visible relations among the samples of a time series. Let ${\mathbb {A}}_m$ denote the adjacency matrix. If there exists a visible relation between two samples, (*i*, *j*), of a time series, then ${\mathbb {A}}_m(i,j)=1$. Otherwise, if an obstacle limits the visibility between two elements (*i*, *k*), then ${\mathbb {A}}_m(i,k)=0$. It should be noted that the immediate neighbors of an the $i^{th}$ element are always visible. By definition, ${\mathbb {A}}_m(i,i) = 0$. Figure 1 depicts this association between the adjacency matrix and the samples of the time series.

The VG theory has been shown to be an appropriate tool for investigating and further determining the structural interrelations among the samples of a time series, which consists of positive defined elements^13^. Inspired by^12^, we have applied VG theory to the SER problem in our previous work^13^, which provided evidence regarding the effectiveness of VG theory in the analysis of speech signals. However, as discussed in^12^, the VG has two disadvantages: first, it does not consider the effect of uneven sampling; second, it cannot capture the time series changes below a zero baseline. Despite that, speech signals usually consist of many samples, i.e., they have a long duration, which makes application of the VG theory difficult. Based on these limitations, the first step of our proposed methodology is to transform each speech signal into another time series amenable to VG transformation.

**Figure 2:**
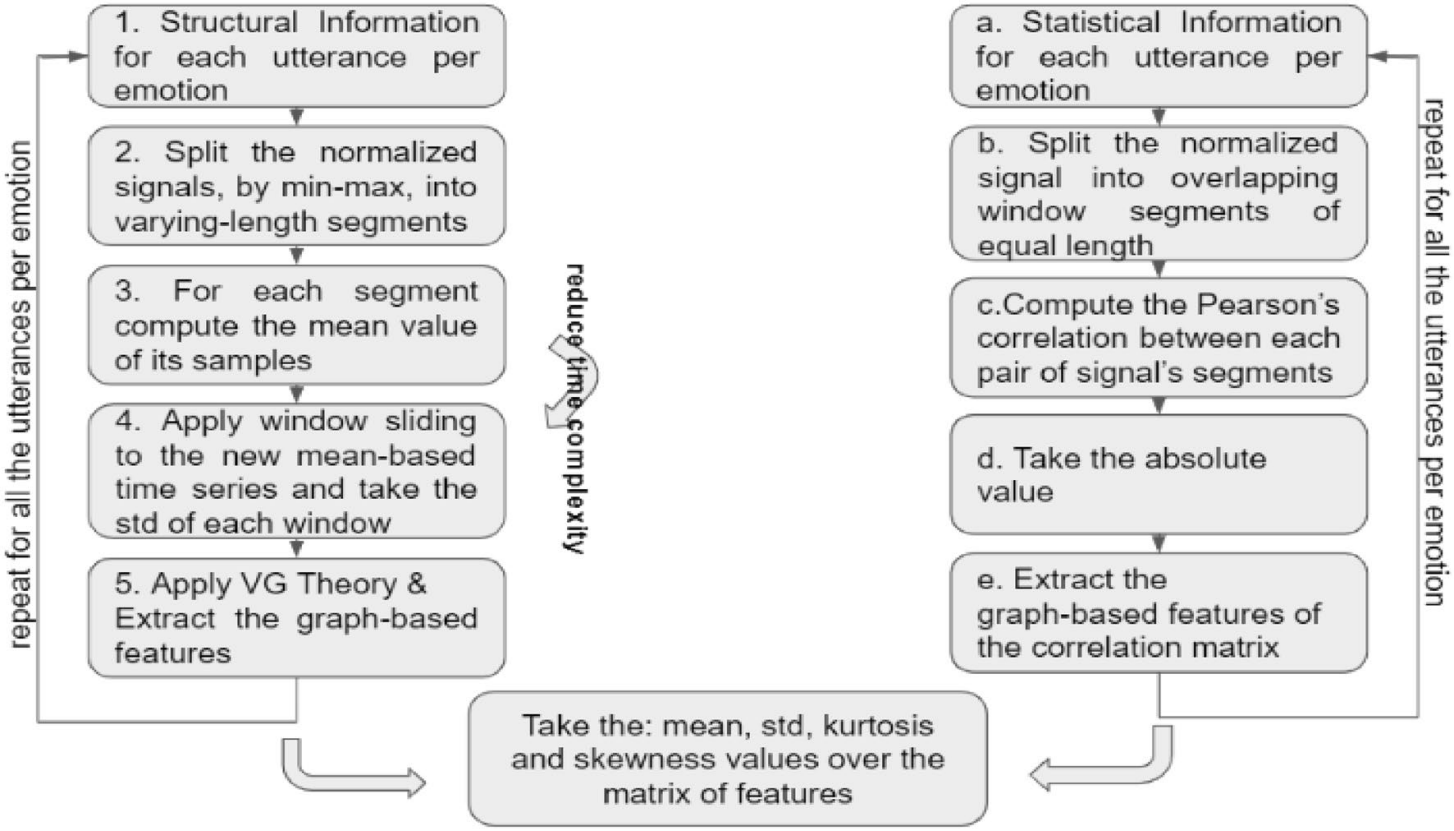
Graph-based speech analysis: (**a**) Exploitation of its structural information (left column); (**b**) Exploitation of its statistical information (right column); (**c**) Concludes to the probabilistic moments computation (i.e., speaker-based emotional motif).

In both mentioned previous works, the signal is segmented into overlapping frames, where the length of each frame is fixed. Although this approach is fast and convenient, important prosodic information in emotional expression may be lost and thus the structural fluctuations of the speech signals may not be fully characterized through the VG analysis^35^ ( Fig.2, step 1). As a consequence, motivated by^35^, we selected varying-length segments, extracted from the Canny, Sobel and Prewitt algorithms^36^ which provide a hierarchical structure, considering the relations of prosodic states. Furthermore, we model the emotional expressions based on syllables, words or sentences^35^ ( Fig.2, step 2). In more detail, through these algorithms we extract the successive points of the time series in which sub-parts of the whole utterance belong, based on the speech structure (comparatively to the edge detection in image analysis). After that, from each segment the mean value over its samples is computed ( Fig.2, step 3). The main reason for employing such an approach is because we want to avoid the high computational cost required when analyzing a long duration time series. Analytically, by retaining the whole ensemble of these samples we increase the VG algorithmic complexity, as, essentially, the VG theory has to pass through the analysis of the whole signal. Hence, the mean value is an appropriate metric for describing the distribution of segments’ samples. It is worth to mention that, each signal has to be normalized based on its maximum and minimum values, so as to take its version to the range of [0, 1], as the Canny, Sobel and Prewitt algorithms require (i.e., grayscale images as inputs) ( Fig.2, step 2).

Computing the mean value of each varying-length segment leads to a new time series, which consists of both positive and negative values. Thus, in order to transfer this time series to a positively defined domain, appropriate as input to the VG procedure, we repeat the sliding windows approach to this new time series. Similarly as in^13^, we split this time series into overlapping windows and also the standard deviation (SD) is further estimated, due to the fact that SD both provides positive values and retains the samples’ variability. It should be noticed that, in terms of our experimental evaluation, the root-mean-square (rms) energy of the signal’s segments was also examined but it was proved less effective than the standard deviation measure. By splitting the mean-value-based already processed utterances into fixed-length segments and taking the standard deviation as a representative value of their samples’ distribution, we achieve to reduce the signals’ length and at the same time to construct a positively defined new time series ( Fig.2, step 4).

The resulting set of time series are now appropriate inputs to visibility graphs. Suppose that we have two sample-points, $x_1$ and $x_2$ which correspond to time indices $n_1$ and $n_2$ of a time series. Then, the visibility between these points is determined by the following geometric criterion:

1 $$\begin{aligned} x_3 < x_1 + (x_2-x_1)\frac{n_3-n_1}{n_2-n_1}\, \end{aligned}$$

where, $(x_3,n_3)$ is every intermediate point such that $n_1< n_3 < n_2$. Simply put, two samples of a given time series cannot “see” each other if there exists an “obstacle” sample between them, i.e., if a sample with greater magnitude blocks the visibility of each other^12^ as defined in Eq. (1) ( Fig.2, step 5).

Overall, the VG theory concludes to an adjacency matrix, i.e., a graph $G_1=(V_1,E_1)$, where $V_1=\{1,\dots , n\}$ is the set of nodes, which in our case represent probabilistic-based parts of the utterances, while the $E_1=\{e_1,\dots , e_m\}$ is the set of edges, i.e., the structural connections of these parts. Subsequently, this adjacency matrix is given as input to the feature extraction procedure, resulting to a vector of the selected graph-based features, which are then used in our analysis as described in section "Graph-based features". It should be noticed that, the adjacency matrix can have a binary or a weighted form. However, in our proposed methodology the binary version gave higher classification performance. The aforementioned steps are summarized in Fig. 2.

### Statistical graph-based speech information

The graph-based theory has many perspectives, including not only the structural analysis of the signals, but also the statistical one. Inspired by the neuroscience field, where researchers take statistical interrelations among the signals of interest to construct a network^37^, we focus in our present work to further investigate whether the statistical information of speech signals could provide a better performance to our pipeline. Consequently, in this section we describe the approach followed so as to create the second important adjacency matrix, which is a result of the statistical interdependencies among segments of an utterance.

Specifically, the first step ( Fig.2, step a) of this analysis is to split each utterance into overlapping windows of fixed-length ( Fig.2, step b) and estimate all the pair-wise Pearson’s correlation between two segments ( Fig.2, step c). Thus, suppose that we have an utterance split into *L* segments. Then, the correlation-based weighted adjacency matrix, of size $(L\times L)$, consists of the Pearson’s interrelation between the $i^{th}$ and $j^{th}$ segments, with $i,j=1,\dots , L$. This leads to a square matrix of both positive and negative values, which essentially describes how the speech signal changes across time, regarding an emotion and a specific speaker. Taking the absolute value of these linear-based strengths, we conclude to the statistical-based quantity, i.e., to a graph defined as $G_2=(V_2,E_2)$ ( Fig.2, step d). In this graph, $V_2=\{1,\dots , n\}$ is the set of vertices, which is equal to the number of the segments, while the $E_2=\{e_1,\dots , e_m\}$ set denotes the linear interdependencies between each pair of the segments, i.e., each $e_i$ denotes a correlation between two segments of length *L*. It should be noted that Pearson’s correlation requires vectors of equal length to estimate their interrelation. To conclude, the last step is to extract the graph-based features described in the next section ( Fig.2, step e). Figure 2 provides a visual representation of all steps of the computational pipeline underpinning our proposed approach.

### Graph-based features

One of the main advantages of graph-based theory is that it can provide a variety of features which characterize the adjacency matrices in a appropriate manner, i.e., one can derive quantitative measures from the adjacency matrix which describe the graph-based representation of a signal. Exploiting this possibility, in our analysis we estimate the following graph-based features: the *degree of connectivity*, the *clustering coefficient*, the *density*, the *averaged value*, the *modularity* and in the case of the structural information the so-called *energy measure*.

In detail, the two basic adjacency matrices, $\mathbf{A_1}, \mathbf{A_2}$, are constructed from the corresponding graphs $G_1, G_2$ describing the structural and the statistical speech information, respectively. Subsequently, from each adjacency matrix corresponding to a single utterance, we extracted the aforementioned features, defined as follows:

- *Degree of connectivity*

As *degree of connectivity* (*DoC*) we denote the number of edges that are immediately connected to a specific node. Formula (2) computes the degree of connectivity measure, as also presented in^38^:

2 $$\begin{aligned} DoC=\sum _{i=1}^N\sum _{j=1}^N\textbf{A}_{i,j}\, \end{aligned}$$

where, $\textbf{A}$ is the adjacency matrix for each case, structural or statistical, and *i*, *j* denote the nodes of the adjacency matrix.

- *Clustering coefficient*

In a graph, as *clustering coefficient* (*CC*) we denote the tendency of a node to create cliques (i.e., clusters). *CC* characterizes the ability of a node to cluster together with other nodes^13^. It takes values to the range of [0, 1], with the better result corresponding to the greater value.

3 $$\begin{aligned} CC_i = \frac{1}{k_i(k_i-1)}\sum _{j=1,l=1}^N\textbf{A}_{i,j}\textbf{A}_{j,l}{} \textbf{A}_{l,i}. \end{aligned}$$

Notice that, the above equation (3) computes the *local* clustering coefficients for an undirected graph. Simply, it is the number of triangles that a node *i* is involved in. As $k_i=\sum _{j=1}^N\textbf{A}_{i,j}$, we denote the number of edges that are connected to node *i*. As a consequence, the *global* clustering coefficient is the mean value over the $CC_i$^13^.

- *Density*

*Density* (*D*) is a graph-based measure which shows how sparse or dense a graph is^13^. As a consequence, a dense graph is has a number of edges close to the maximal number of edges. Its computation derives from the following equation:

4 $$\begin{aligned} D=\frac{2|E|}{N(N-1)}\, \end{aligned}$$

where as |*E*| we denote the number of the edges included in a graph of *N* number of nodes.

- *Averaged value*

An also effective feature, which proved to raise the classification accuracy in terms of our analysis, is the *averaged value* (*M*), computed as the mean value over the elements of the chosen adjacency matrix.

- *Modularity*

*Modularity* (*Q*) is a graph measure which describes the graph’s strength of division, i.e., its tendency to be split into clusters, the so-called modules^12^. A high modularity value characterizes a graph with a tied structure. Usually, the connectivity in a module is strong, whereas between two modules weak. In more detail, modules in a graph structure are communities of edges grouping together the corresponding nodes. This implies that, a high modularity value mathematically is interpreted as a community in which more edges exist than it is expected by chance.

Modularity is computed based on the following formulas, as described in Ref.^12^:

5 $$\begin{aligned} Q = \sum _i^c (\textbf{e}_{ii}-a_i^2)\, \end{aligned}$$

where, $e_{ij}$ is the fraction of edges with the one end in the belonging to the community, denoted as *c*, *i* while the other to the community *j*, computed by:

6 $$\begin{aligned} {\textbf{e}}_{ij} = \sum _{u,v} \frac{\textbf{A}_{u,v}}{2m}\textrm{1}_{u\in c_i}\textrm{1}_{v\in c_j} \end{aligned}$$

and $a_i=\sum _j \textbf{e}_{ij}$. Finally, *m* is equal to the number of links.

- *Energy measure*

We also selected a graph-based quantity derived from the decomposition of the adjacency matrix, the so-called *energy measure* (*E*) of a graph^39^. Based on^39^, the energy measure is defined as follows:

7 $$\begin{aligned} E = \sum _i\lambda _i^2, \end{aligned}$$

where as $\lambda$’s we denote the eigenvalues estimated through the eigendecomposition of an adjacency matrix, i.e., essentially, the graph spectrum. The energy measure was estimated from the adjacency matrix $\mathbf{A_1}$ which corresponds to the structural information of the signal. We also calculated *E* from the statistical-based adjacency matrix ($\mathbf{A_2}$). However, using this feature did not increase our experimental performance.

Overall, six (6) graph-based features were computed from the structural-based adjacency matrix, and five (5) additional, statistically-based features were also computed.

### Speaker-based emotional motif

A common problem in the analysis of most of the databases is their imbalance, which mostly refers to the unequal number of elements belonging to the classes. The most prominent approach of overcoming this problem, from the ML perspective, is through computing the Unbalanced Accuracy Ratio (UAR)^5^. UAR accuracy is a function of the true-positive to the false-positive rates, i.e., to the sensitivity^5,9^. However, by averaging the class-wise recalls implies that each class contributes to the extracted accuracy with the same probabilistic weight, even though the sensitivity is analogous to the class elements. Thus, researchers usually strengthen their UAR-based results by accompanying them with the $F_1$-Score and the specificity^40^.

On the other hand, recently, studies have focused alternative solutions, such as the data augmentation^41^. Data augmentation is a process of artificially increasing the amount of data by generating new data points from existing data. Although such approaches are effective in many experimental cases a major limitation exists regarding SER: i) in SER we focus on emotion recognition from voice signals, i.e. to address the problem in a speaker-independent manner. Moreover, in small datasets, which sometimes consist of few utterances per emotion and per speaker, it is difficult to create a large number of copies to overcome the imbalance problem.

Furthermore, the imbalance problem also refers to the length of the (speech) signals. Regarding SER, the unequal length of the utterances affects the proposed models, leading to the most prominent solutions, i.e., to the zero-padding or to the limitation of the signals to the minimum possible length. However, in both approaches we take biased classification results^42^.

In overcoming the problems mentioned before, we propose the “speaker-based emotional motif”. Specifically, we compute the first four probabilistic moments over the number of the available utterances per emotion and per speaker, motivated by the GeMAPS mathematical concept. Analytically, suppose that an emotional group consists of *m* utterances, then we estimate mean, standard deviation, skewness, and kurtosis, for each of the 11 graph-based features employed, $F_i\in {\mathbb {R}}^m$.

1. $$\displaystyle \mu = \frac{1}{m}\sum _{j=1}^m F_i(j)$$
2. $$\displaystyle \sigma = \sqrt{\frac{1}{m}\sum _{j=1}^N(F_i(j)-\mu )^2}$$
3. Skewness $$\displaystyle = {\mathbb {E}}\left[ \left( \frac{F_i-\mu }{\sigma }\right) ^3\right]$$
4. Kurtosis $$\displaystyle = {\mathbb {E}}\left[ \left( \frac{F_i-\mu }{\sigma }\right) ^4\right]$$

Finally, we compute 44 quantities which characterize each speaker’s emotion in a unique manner, providing them an individual probabilistic graph-based identity, i.e., 6 structural graph-based features and 5 statistical graph-based features concluding to 4 statistic measures over them (i.e., 44 in total).

## Experimental evaluation

In this section we present important information, firstly, about the datasets used. After that, we analyze the comparative methods, existing in the literature, which essentially provide us the baseline results. Finally, we conclude to our experimental procedure and performances.

### Data description

As this is an extended analysis of the application of the graph-based theory to the SER field, we analyzed two well-known public databases, the Berlin Database of Emotional Speech (EMODB), the Greek Database Aced Emotional Speech Dynamic Database is a Speech Emotion Recognition Dataset (AESDD) and the Database of Elicited Mood in Speech (DEMoS). Analytically, the main characteristics of these databases are presented next:

- *EMODB*: Recordings consist of German speech specific sentences and are made by actors. Specifically, 5 male and 5 female subjects acted 7 emotions, anger, disgust, anxiety, boredom, happiness, sadness and neutral. The ages of the actors belonged to the range of $21-35$ years. In total, it consists of 535 utterances^43^.
- *DEMoS*: This database includes 9365 Italian speech utterances, produced in a realistic manner, i.e., they contain in-the-wild context. In terms of our analysis, we evaluated a subset of the available subjects, i.e., we employed 21 females and 33 males which involve almost 6500 utterances. DEMoS describes the “big six” emotional states, i.e., the anger, sadness, happiness, fear, surprise and disgust, plus the “guilt” emotional state^11^.
- *AESDD*: This database includes almost 600 Greek speech utterances, produced in an actor-based manner. It contains recordings which describe five primary emotions, i.e., anger, disgust, fear, happiness and sadness. It is important to mention that the accuracy of human listeners was estimated at around $74\%$.^44,45^.

It should be noticed that, the EMODB data was recorded at 48 kHz and then down-sampled to 16 kHz. Thus, we up-sampled the recordings again at the initial sampling frequency to take comparative results to the DEMoS, which was recorded at 44 kHz. Both databases are highly imbalanced, whereas the AESDD is balanced.

### Comparative methods

In order to perform a comparative evaluation of the results obtained from our analysis, we decided to compare our methodology with two well-established SER analysis methods. Namely, we compared our results with i) those obtained when sets of handcrafted acoustic features are employed, combined with standard ML approaches, and ii) those obtained when a state-of-the-art DL architecture combined with spectrograms is used. More specifically, for the feature extraction method we employed the eGeMAPS set. The eGeMAPS set comprises 88 acoustic parameters in total. It should be mentioned that, the eGeMAPS use the mean value and the standard deviation probabilistic moments over the selected features extracted from each signal segment^5^.

**Figure 3:**
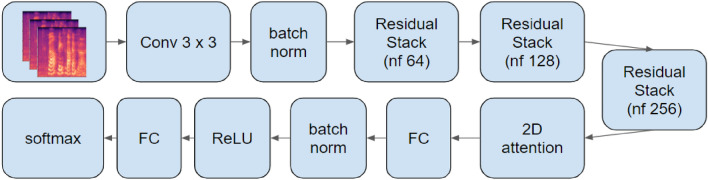
DL architecture proposed in Ref.^9^. Gerczuk et al. use the Mel-Spectrograms of the speech signals as inputs to a ResNet to classify and recognize the emotions of each dataset.

Regarding (ii), we combined the Mel-Spectrograms with a ResNET. Mel-Spectrograms are derived from the audio representation through spectrograms. They apply dimensionality reduction to the log-magnitude spectrum based on the mel-filter^9^. After that, the spectrogram figures pass through a Residual Network (ResNet) which consists of a variety of decomposition steps, as these are presented in Fig. 3. The extraction of the spectrograms is a state-of-the-art approach of processing speech signals^18^. This proposed RenNet contains the following blocks: The first block consists of a concolutional layr with 32 filters of size $3\times 3$. These filters are convolved with the input spectrograms using a stride of 1. The output passes through a batch normalization procedure. Afterwards, the output passes through three consecutive submodules, which use 64, 128 and 256 filters of shape $3\times 3$, respectively. Additionaly, a convolutional layer with a stride of 2 is followed by a batch normalization and cocnludes to a Rectified Linear Unit (ReLU) activation. Then, a convolutional layer of stride 1 and a batch normalization are applied to the output. In order to return to the first block size, an average pooling with patches $2\times 2$ and stride 2 is applied. Finally, the network concludes with a Softmax as well as a fully-connected classification layer.

### Experimental results

In the experimental evaluation of our proposed methodology we examined two databases, EMODB and DEMoS, that are widely used for SER research. Regarding the hyper-parameters necessary in our methodology, i.e., the window’s length and the overlap, we examined a variety of parameters and those selected are presented in Table 1. These values gave the highest performance of our approach and they are analogous to the duration of each database utterances, implying that, for long duration speech signals the approach requires larger window length and thus overlap.

**Table 1:** Hyper-parameters most appropriate values (in samples).

Information	(Samples)	EMODB	DEMoS	AESDD
Statistical	Window	10000	8000	8000
	Overlap	2000	6000	6000
Structural	Window	200	100	200
	Overlap	50	50	100

In Table 2 the results of our proposed methodology are presented, together with those obtained when applying the two, previously described, comparative estimation methods, i.e., the eGeMAPS combined with a Random Forest classifier, of 500 trees with the default Matlab option of “classification” method, and the Mel-Spectrograms with a ResNET.

**Table 2:** Classification LOSOCV Accuracy $[\%]$.

Methodology	Metric	EMODB	DEMoS	AESDD
eGeMAPS	UAR	48.4^9^	41.19	57.1^9^
Mel-Spec. and ResNet	UAR	59.8^9^	72.76	37^9^
Graph-based	Speaker-based	${77}.{8}$	${79}.{1}$	$70$

For classification we used a “leave-one-speaker-out-cross-validation” (LOSOCV) approach, based on relevant literature and specifically the study in^9^. This implied that at each experimental classification we retain one speaker for testing while the rest were used for training. This procedure was repeated for all the available speakers.

Concerning the use of the eGeMAPS set of features, we employed different ML classifiers. Namely, we used the support vector machine (SVM) classifier for multiclass problem, i.e. for the recognition of emotions present in the EMODB database, whereas the random forest classifier proved to be more effective for the classification of the emotions present in the DEMoS database. Regarding the Mel-Spectrograms combined with the ResNet architecture methodology, we evaluated the DEMoS database by repeating the experimental procedure for the selected sub-set of the DEMoS. In both experimental evaluations the classification accuracy was the UAR, to overcome the datasets’ imbalance problem^46^. In the case of the AESDD analysis and evaluation, the selected classification method was the Random Forest of 300 tree length.

Observing the results presented in Table 2, it became evident that our graph-based methodology combined with the speaker-based motif achieved the highest classification performance for both databases, with the use of the RF classifier. Analytically, for the EMODB we managed to significantly increase the classification accuracy, which reached $77.8\% (\pm 16\%)$ (standard deviation). The performance of our proposed approach on the DEMoS was also important as it was the highest among the comparative methods and equal to $79.1\% (\pm 20\%)$ (standard deviation). Regarding the AESDD, the performance reached the $70\% (\pm 20\%)$ (standard deviation).

It is of major importance to mention that, when we applied the speaker-based motif on the eGeMAPS set of features, i.e., we computed the set of features per utterance and then we estimated the mean value and the standard deviation over the number of the available utterances per emotion and per speaker, the accuracy was increased by almost $20\%$. Moreover, regarding the “Metric” mentioned in Table 2, we would have to clarify that the UAR accuracy estimated from multiple utterances equals to the speaker-based motif when we essentially have “one” utterance. In more detail, the sensitivity measure gives the same results.

Overall, our proposed graph-based procedure accomplished by the speaker-based motif for the classification outperformed all the methods assessed on the selected databases.

**Figure 4:**
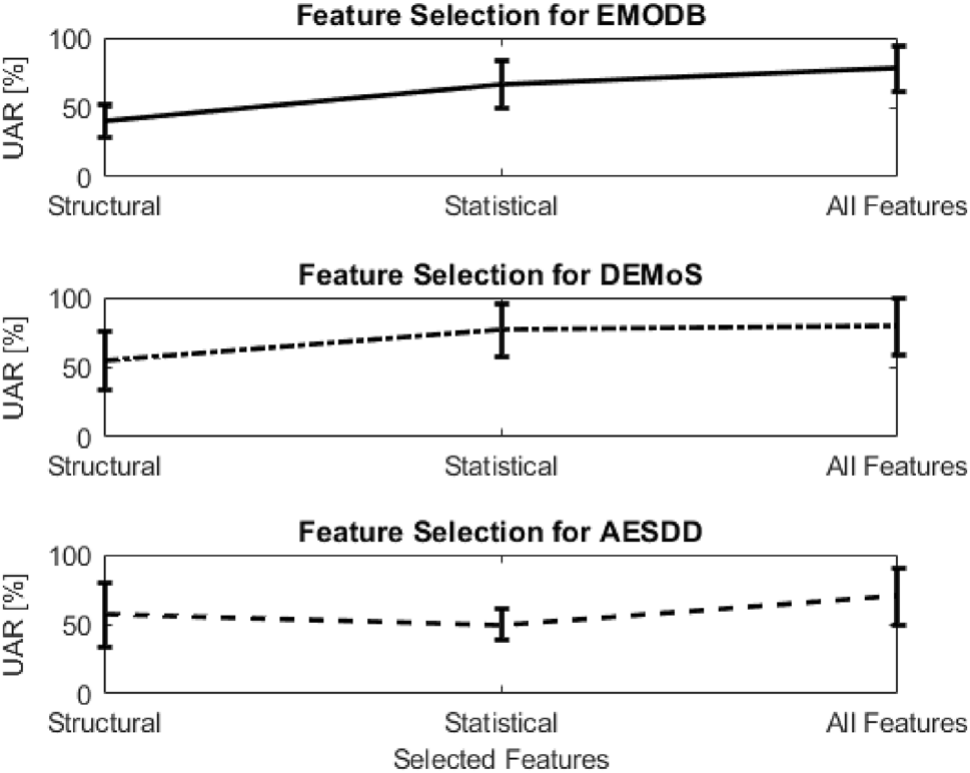
Feature Selection for all the Databases.

**Figure 5:**
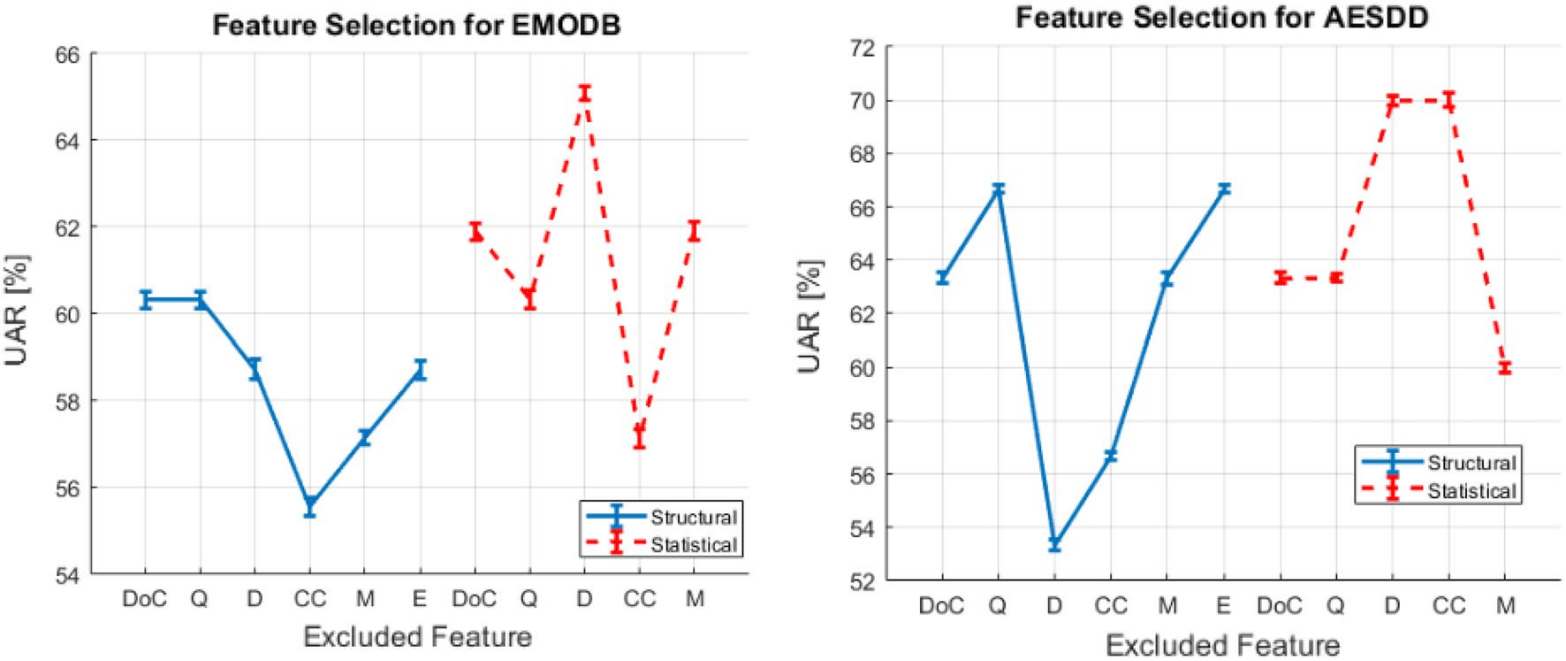
Graph-based Feature Exclusion-Selection for EMODB (left figure); Graph-based Feature Exclusion-Selection for AESDD (right figure).

Finally, through our experimental analysis, we used two different feature selection methods. The first one concerned the comparison of the selection of (i) the structural graph-based features, (ii) the statistical graph-based features and (iii) both categories. As it is presented in Fig. 4, EMODB and DEMoS database appear to have a similar classification UAR accuracy, with the statistical graph-based features to be more prominent than the structural ones. On the other hand, there seems to be less trend towards this direction for the AESDD database analysis. Moreover, regarding the second evaluation, we passed through the examination of all the graph-based features and each one of them affects the pipeline’s performance. Specifically, as we can observe in Fig. 5, we took a similar UAR classification performance for the two examined databases, i.e., the EMODB and the AESDD (the same behavior was for the DEMoS, too). Significantly, among the most important graph-based features seem to be the structural-based density (D) and the clustering coefficient (CC). This probably implies that the more the voice fluctuates the denser the speech graph appears to be, and thus more cliques (clusters), i.e., higher D and CC values, are present in the graph. To conclude, if we exclude the density and clustering coefficient structural graph-based features, the performance of our pipeline decreases.

## Discussion

Speech emotion recognition is a complicated computational problem as the emotions are difficult to be analyzed and recognized by humans. As a consequence, advanced mathematical analyses are required so as to achieve recognizing the primary emotional states. In our proposed methodology, we combined two important theories, i.e., the graph-based and the machine learning possibilities. Specifically, the graph-based theory is among the most important mathematical tools which provides many signal processing perspectives. Exploiting the statistical information of speech, on the one hand, and the structural material of the utterances, on the other, through simple, fast and accurate mathematical background, we constructed a novel pipeline for the analysis of voice signals. Moreover, well-known graph-based features were extracted from each of the two directions (i.e., the statistical and the structural), which describe the speech signals’ graph representations.

Furthermore, our analysis revealed that exploiting the statistical measures over the graph-based features, which represent the speaker’s utterances, is more effective than retaining the whole ensemble of utterances. This motif, denoted as speaker-based emotional motif, provides a unique emotional identity to each speaker’s emotional state and by evaluating this manner through well-established machine learning procedures, proved to help the classifier’s model to recognize the emotional states in a more accurate manner.

Our proposed methodology was compared with state-of-the-art methodologies, i.e., utilizing the eGeMAPS parameter set or employing Mel-spectrograms analysis through DL architectures. In all the experimental scenarios, our pipeline proved to be more effective in recognizing the examined emotional states, not only on actor-based data but also on context-free speech signals. It is worth to notice that, evaluating the speaker-based emotional motif on the variety of the eGeMAPS feature set, the classification accuracy was raised significantly. Overall, our methodology proved to be more accurate than the existing implementations, through a simple, fast and novel approach.

Regarding feature selection, we observed that among the most important graph-based features are the density and the clustering coefficient which were derived from the structural-based analysis of the speech. Finally, the structural-based combined with the statistical-based graph features proved to be important in our analyses.

Overall, our proposed pipeline proved to be more effective than the comparative methods and has the following advantages: (i) the graph-based theory gives the opportunity to the researchers to analyze the speech signals by taking structural and statistical interrelations among the speech signal windows, (ii) through our pipeline we achieved to exploit two important signal processing information, i.e., the geometrical voice structures as well as the statistical ones, (iii) important graph-based features were employed in our analysis, proving their superiority over the traditional and well-established features, (iv) the ML-based analysis of three databases proved to be more effective than well-established DL-based approaches, and (v) the introduction of the first four statistical moments on our pipeline, led to the speaker-based motif classification which gave an important boost to our experimental performance.

## Conclusions

In this study we applied the graph-based theory on the SER field. Specifically, extending our previous work, we combined the structural and statistical information of speech signals. Exploiting these graph-based quantities, we created an innovative, speaker-based motif of classification. In more detail, by using basic statistics over the utterances we created a unique emotional “identity” for each speaker, which proved to be more effective than the utterance-based previously used motif.

To conclude, through our proposed methodology we achieved to the following goals: (i) successfully employ the graph-based theory on speech emotion recognition, both in actor-based and in context-free speech recordings, (ii) use a small number of graph-based features, which significant reduces the computational requirements, (iii) create a speaker-based classification motif in contrast to the utterance-based motif that is usually employed, and (iv) achieve the highest performance to date (on the EMODB, AESDD and DEMoS databases). Specifically, we observed an average UAR increase of almost $18\%$, $8\%$ and $13\%$, respectively, for these three datasets.

In the future, we aim to evaluate our proposed method on speech or mental disorders, such as the depression^47,48^. Furthermore, we would like to evaluate not only speech-based data but also visual ones, as already presented in existing works^49–51^.

## Data Availability

1. The EMODB dataset analyzed during the current study is publicly available in the Kaggle repository,https://www.kaggle.com/datasets/piyushagni5/berlin-database-of-emotional-speech-emodb. 2. The DEMOS dataset analyzed during the current study is publicly available in the Zenodo repository, https://zenodo.org/record/2544829, after signing the appropriate End User License Agreement (EULA). 3. The AESDD dataset analyzed during the current study is publicly available in the http://m3c.web.auth.gr/research/aesdd-speech-emotion-recognition/.
